# Potassium measurements and risk of type 2 diabetes: a dose-response meta-analysis of prospective cohort studies

**DOI:** 10.18632/oncotarget.21823

**Published:** 2017-10-11

**Authors:** Yang Peng, Guo-Chao Zhong, Qiao Mi, Kejia Li, Ao Wang, Ling Li, Hua Liu, Gangyi Yang

**Affiliations:** ^1^ Department of Endocrinology, The Second Affiliated Hospital, Chongqing Medical University and Chongqing Clinical Research Center for Geriatrics, Chongqing, China; ^2^ Department of Hepatobiliary Surgery, The Second Affiliated Hospital, Chongqing Medical University, Chongqing, China; ^3^ Key Laboratory of Diagnostic Medicine (Ministry of Education) and Department of Clinical Biochemistry, College of Laboratory Medicine, Chongqing Medical University, Chongqing, China; ^4^ Department of Pediatrics, University of Mississippi Medical Center, Jackson, Mississippi, USA

**Keywords:** potassium, type 2 diabetes mellitus, systematic review, meta-analysis, dose-response

## Abstract

**Objective:**

To clarify the relationship between serum, dietary, and urinary potassium and the risk of type 2 diabetes mellitus (T2DM).

**Materials and Methods:**

We searched PubMed and EMBASE through January 6, 2017 for studies reporting risk estimates on the association of potassium measurements and the risk of T2DM. The summary risk estimates were obtained through a random-effects model. Dose-response analysis was conducted.

**Results:**

Eight studies involving 5,053 cases and 119,993 individuals were included. A trend toward significance was found in the highest versus lowest meta-analysis on serum potassium and T2DM risk (RR = 0.79; 95% CI 0.60–1.04); moreover, the RR per 1 mmol/L increase in serum potassium was 0.83 (95% CI 0.73–0.95). A non-significant association of dietary potassium and T2DM risk was detected (RR for the highest versus lowest category: 0.93; 95% CI 0.81–1.06; RR for every 1000mg increase per day: 1.00, 95% CI 0.96–1.05). A similar non-significant association was found for urinary potassium and T2DM risk (RR for the highest versus lowest category: 0.83; 95% CI 0.39–1.75; RR per 10 mmol increase: 1.00; 95% CI 0.95–1.05). Evidence of a linear association between serum, dietary, and urinary potassium and the risk of T2DM was found (all P_non-linearity_ > 0.05).

**Conclusions:**

Low serum potassium increases the risk of T2DM in a linear dose-response manner; nevertheless, neither dietary potassium nor urinary potassium shows any association with the risk of T2DM. However, these findings should be interpreted with caution due to limited studies.

## INTRODUCTION

According to the pooled data of the International Diabetes Federation from 219 countries and territories, diagnosed diabetes reached 381.8 million cases in 2013 and is projected to reach 591.9 million by 2035 [1]. Accounting for 90% of all diabetes cases, type 2 diabetes mellitus (T2DM) is determined by both genetic and environmental factors [2]. Traditional risk factors, including sedentary habits, high-fat diets and obesity, have been widely accepted [2–4]. In addition, emerging investigations have found a spectrum of biomarkers in relation to the risk of T2DM, including interleukin-6, C-reactive protein, thyroid-stimulating hormone and serum selenium [5–8].

Potassium is a chief cation in the intracellular fluid and plays a critical role in water- electrolyte balance. In the past decade, some large scale epidemiological studies have explored the association between serum, dietary, and urinary potassium and the risk of T2DM [9–14]. However, the relationship between potassium measurements and T2DM risk still remains controversial. Several studies have suggested that higher serum potassium levels are related to a lower risk of T2DM [10, 13–15]. However, this inverse association has not been observed in other studies [9, 11] regarding the association of serum potassium with the risk of T2DM. Similarly, several studies regarding dietary and urinary potassium showed that low potassium intake was related to an increased risk of T2DM [12, 16], while other studies found non-significant results [10, 11, 14, 17]. Moreover, the nature of the dose-response association between potassium measurements and T2DM risk remains unknown.

To our knowledge, a systematic review and dose-response meta-analysis of potassium measurements and T2DM risk is not currently available. Therefore, we conducted this study to clarify the relationship between serum, dietary, and urinary potassium and the risk of T2DM.

## RESULTS

### Literature search

**Figure 1:**
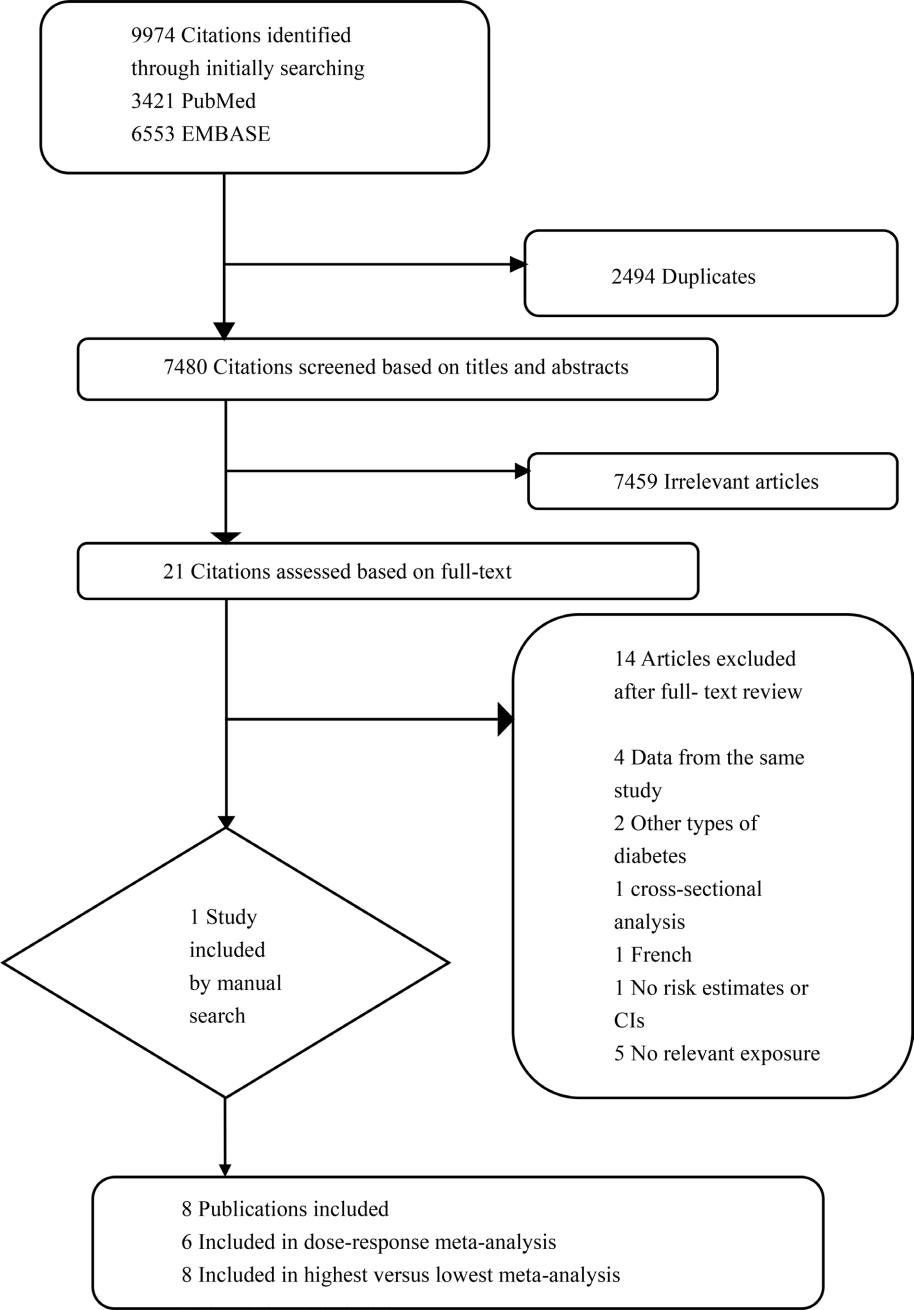
Flowchart for the selection of studies

As shown in the flow chart (Figure 1), 7,480 citations were identified from PubMed and EMBASE after removing duplicates. A total of 7,459 obviously irrelevant citations were excluded by scrutinizing titles and abstracts. Fourteen citations were further excluded after carefully reading of the full text according to several reasons described in Figure 1. One study was included after manual search. Finally, eight studies were included in this meta-analysis for comparing highest versus lowest category, six of which were eligible for the dose-response meta-analysis.

### Study characteristics

The characteristics of the eight identified studies are summarized in Table 1. Our meta-analysis involved 5,053 cases and 119,993 individuals. All studies were large prospective cohort studies, most of which were conducted in the USA, besides TOPICS 1 [13] in Japan and a study in Finland [17]. Except TOPICS 1 study [13] which recruited male subjects from Japanese government employees and NHS [16] which recruited female registered nurses, the remaining six were community-based studies that were conducted in the general population comprised of both men and women. The follow-up durations were from 5 to 18.1 years with a baseline age range from 18 to 95 years. The incident diabetes cases were ascertained based on the following items across studies: (1) A fasting blood glucose level ≥ 126mg/dL (7 studies); (2) A non-fasting glucose ≥ 200 mg/dl (5 studies); (3) The glycated hemoglobin ≥ 6.5% [to convert to proportion, multiply by 0.01] (3 studies); (4) The use of glucose-lowering medications (5 studies); (5) Participants self-reported clinician-diagnosed diabetes (2 studies). Serum potassium was measured using the ion-selective electrode method. Dietary potassium was estimated from food frequency questionnaire (FFQ). Urinary potassium samples were analyzed by potentiometric methods. Most of the included studies provided risk estimates adjusted for age, sex, race, BMI and family history of diabetes.

**Table 1:** Characteristics of the studies included in the meta-analysis

Author year	Gender Age (years)	Country Duration (years)	Potassium category	Sample size (cases)	Diabetes ascertainment	Adjustment
Chatterjee^9^ 2016	M&F 45–84	US 8	Serum K^+^	5415 (1281)	FBG ≥ 126mg/dL use of DM medications	Age, sex, race, study site, waist circumference, BMI, smoking, family history of DM, education, income, alcohol use, physical activity, systolic blood pressure, antihypertensive medication use and use of other medications + total energy intake (for dietary K^+^)
			Dietary K^+^	5415 (1281)		
Chatterjee^10^ 2016	M&F 21–95	US 8	Serum K^+^	2157 (398)	FBG ≥ 126 mg/dL HbA1c ≥ 6.5% use of DM medications	Age , sex, BMI, waist circumference, serum sodium, creatinine, physical activity, family history of DM, presence of hypertension, systolic blood pressure, use of diuretics, fasting glucose and insulin, and income. + total calorie intake, dietary fat intake, saturated fat intake, fiber intake, and dietary sodium intake, total fruit and vegetable intake. (for dietary K^+^) + urinary spot creatinine, urinary spot creatinine, aldosterone (for Urinary K^+^ )
			Dietary K^+^	1999 (367)		
			Urinary K^+^	1376 (227)		
Chatterjee^11^ 2015	M&F ≥ 65	US 12	Serum K^+^	4754 (445)	FBG ≥ 126 mg/dL nonfasting glucose ≥ 200 mg/dL use of DM medications	Age, sex, race, clinic site, BMI, waist circumference, physical activity, smoking, alcohol use, systolic blood pressure, and use of ACEI, beta blockers, and diuretics + diet score and total energy intake (for dietary K^+^)
			Dietary K^+^	4111 (375)		
Chatterjee^12^ 2012	M&F 18–30	US 12.9 /16.8	Dietary K^+^	4754 (373)	FBG ≥ 126mg/dl 2 hPG ≥ 200 mg/dl HbA1c ≥ 6.5% use of DM medication	Average of three 24 h urinary creatinine measures, age, sex, race, BMI, family history of DM, systolic blood pressure, physical activity level and education level (for Urinary K^+^ )
			Urinary K^+^	1066 (99)		
Heianza^13^ 2011	M 25–80	Japan 5	Serum K^+^	4409 (250)	FBG ≥ 7.0 mmol/l HbA1c level ≥ 6.5% clinician-diagnosed DM	Age, parental history of DM, BMI, hypertension, HDL cholesterol, triacylglycerol and smoking habit, HbA1c, FBG
Chatterjee^14^ 2010	M&F 45–65	US 9	Serum K^+^	12209 (1475)	FBG ≥ 126mg/dL nonfasting glucose ≥ 200 mg/dL clinician-diagnosed DM use of DM medications	Age; sex; race; center; BMI; waist circumference; serum magnesium, calcium, and creatinine levels; physical activity levels; parental history of DM; presence of hypertension; systolic blood pressure; FBG and fasting insulin levels; income; and use of beta blockers, diuretics, ACEI. +dietary magnesium intake, dietary calcium intake (for dietary K^+^)
			Dietary K^+^	11530 (NA)		
Hu^17^ 2005	M&F 35–64	Finland 18.1	Urinary K^+^	1935 (129)	diagnosis of DM on the basis of the World Health Organization criteria	Age, sex, study year, education, physical activity, smoking status, alcohol consumption, coffee consumption, vegetable consumption , fruit consumption , sausage consumption, bread consumption ,saturated fat consumption, systolic blood pressure, antihypertensive drug , BMI
Colditz^16^ 1992	F 34–59	US 6	Dietary K^+^	84360 (702)	symptom + FBG ≥ 7.77 mmol/L a random BG ≥ 11.1 mmol/L at least two BG ≥ 11.1 mmol/L	Age, BMI, alcohol intake, family history of DM, prior weight change, time period.

Abbreviation: ACEI , Angiotensin-Converting Enzyme Inhibitors; BG , blood glucose; BMI , Body Mass Index; Dietary K^+^, Dietary potassium; DM , diabetes mellitus; FBG , fasting blood glucose; HbA1c , glycosylated hemoglobin; HDL , high density lipoprotein; M&F , Male and Female; NA , Not available; Serum K^+^ , serum potassium; US , United States; Urinary K^+^ , Urinary potassium; 2hPG , 2-hour postprandial blood glucos

### Quality assessment and publication bias

When it came to quality assessment, six studies had 7 or 9 stars, whereas the remaining two had 5 stars, indicating the quality of included studies is generally good (Supplementary Table 1). In addition, no evidence of publication bias was detected by Egger’s and Begg’s tests (all *P* > 0.05; Supplementary Figures 1–3).

### Serum potassium and T2DM

**Figure 2:**
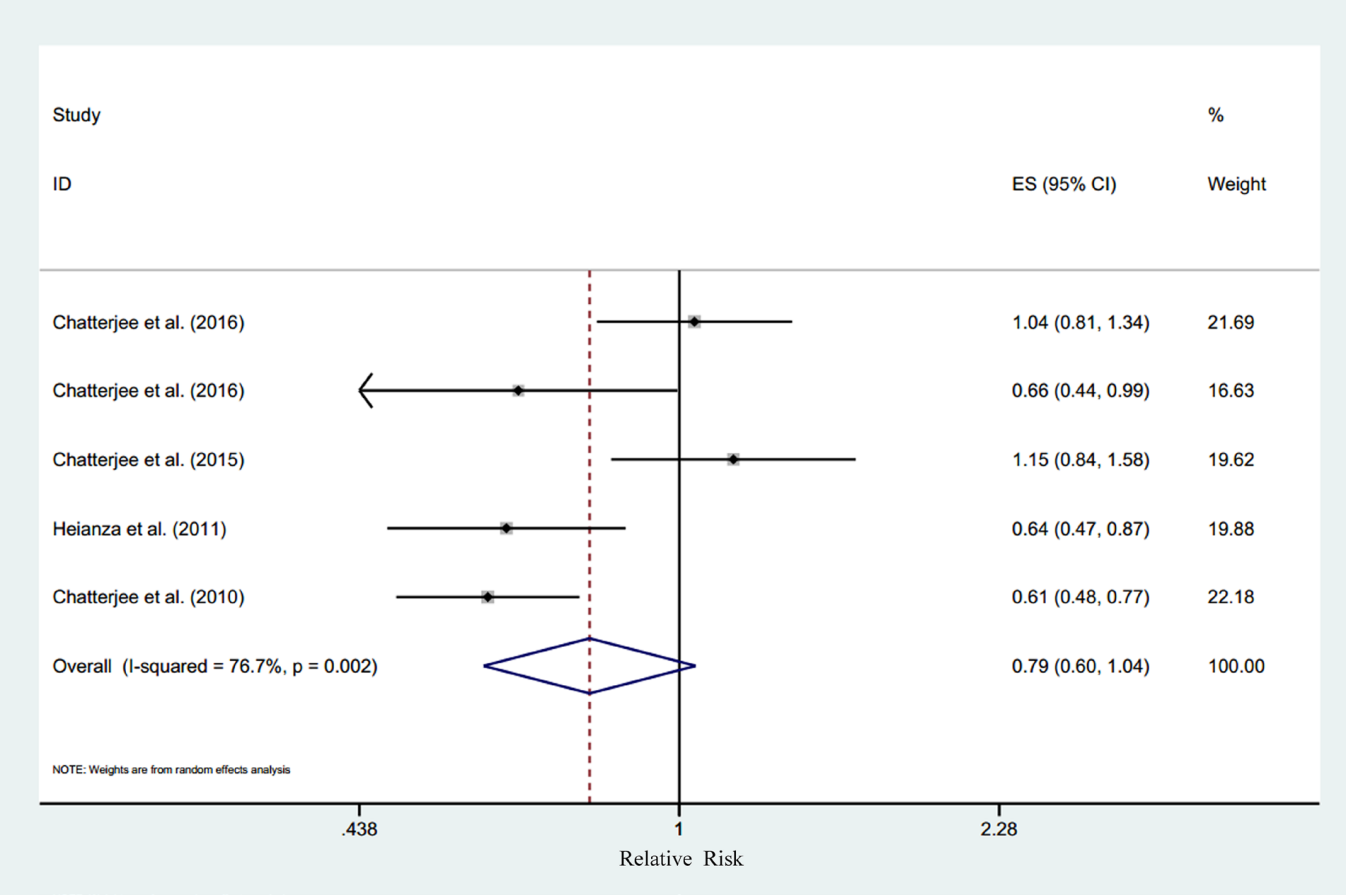
Relative risk of type 2 diabetes according to the highest vs. lowest category of serum potassium Note: CI = confidence interval, ES = effect size.

**Figure 3:**
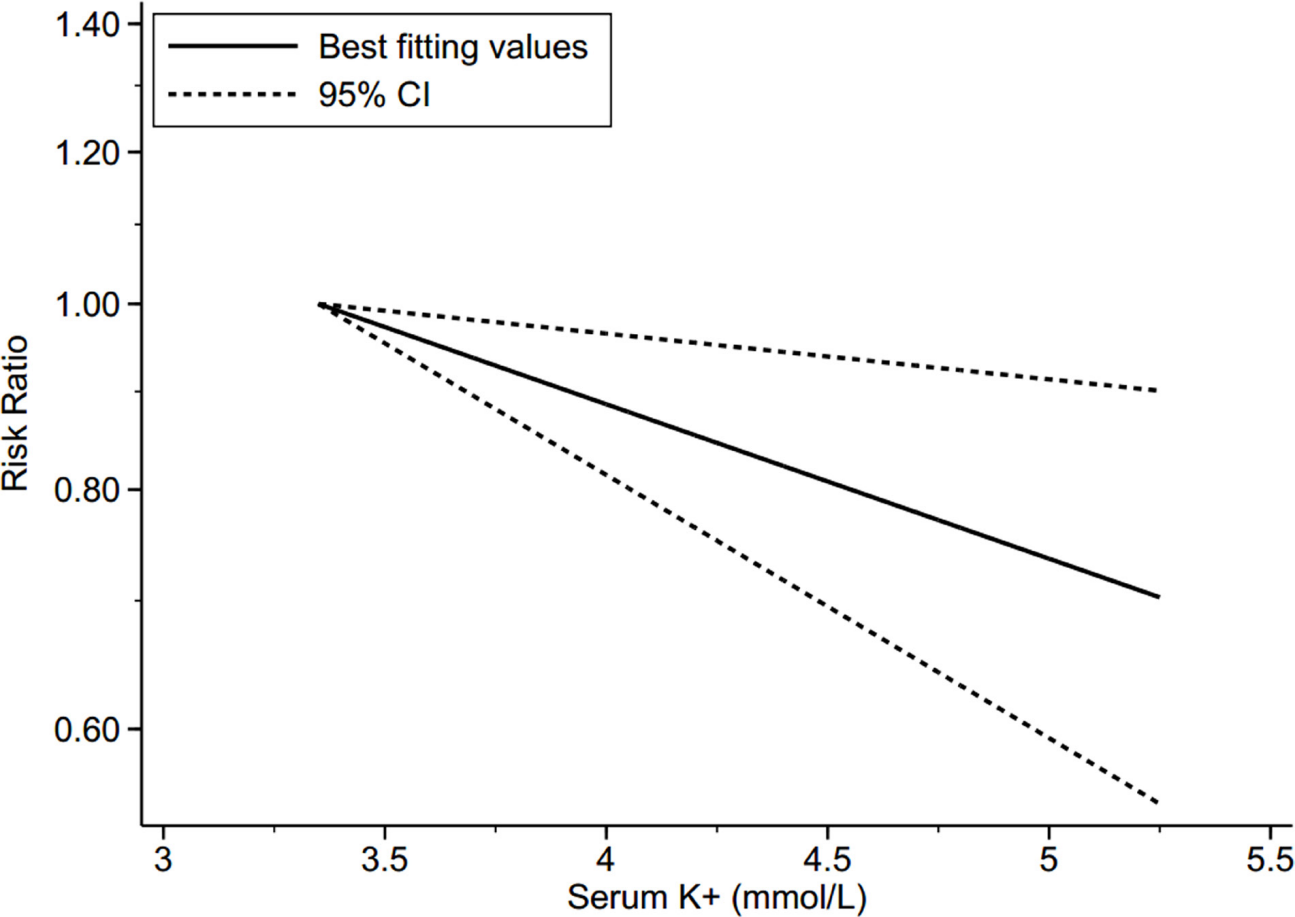
Dose-response relationship between serum potassium and risk of type 2 diabetes Note: Risk ratio indicates the relative risk of type 2 diabetes. CI = confidence interval, Serum K^+^ = serum potassium.

Five studies [9–11, 13, 14] were included in the analysis of serum potassium and the risk of T2DM, involving 28,944 individuals and 3,849 T2DM cases. The summary RR for the highest versus lowest meta-analysis was 0.79 (95% CI 0.60–1.04), with substantial heterogeneity (*P*_heterogeneity_ < 0.01, I^2^ = 76.7%, 5 studies) (Figure 2). Through sensitivity analysis, a significant inverse association between serum potassium and T2DM risk (RR = 0.63, 95% CI 0.52–0.73) with a remarkably decreased level of heterogeneity (*P =* 0.94, I^2^ = 0%) was found by ignoring two studies [9, 11] with much older mean age. The mean age of the participants in the 3 studies included [10, 13, 14] was under 55 years, while in the 2 studies taken out [9, 11] the mean age was over 60 years (Supplementary Figure 4). The random dose-response meta-regression model showed that the RR per 1 mmol/L increase in serum potassium was 0.83 (95% CI 0.73–0.95; goodness-of-fit χ^2^ = 18.59, *P*_goodness-of-fit_ = 0.07, 5 studies). We found evidence of a linear trend (*P*_non-linearity_ = 0.10) by using restricted cubic spline model [18] (Figure 3).

### Dietary potassium and T2DM

**Figure 4:**
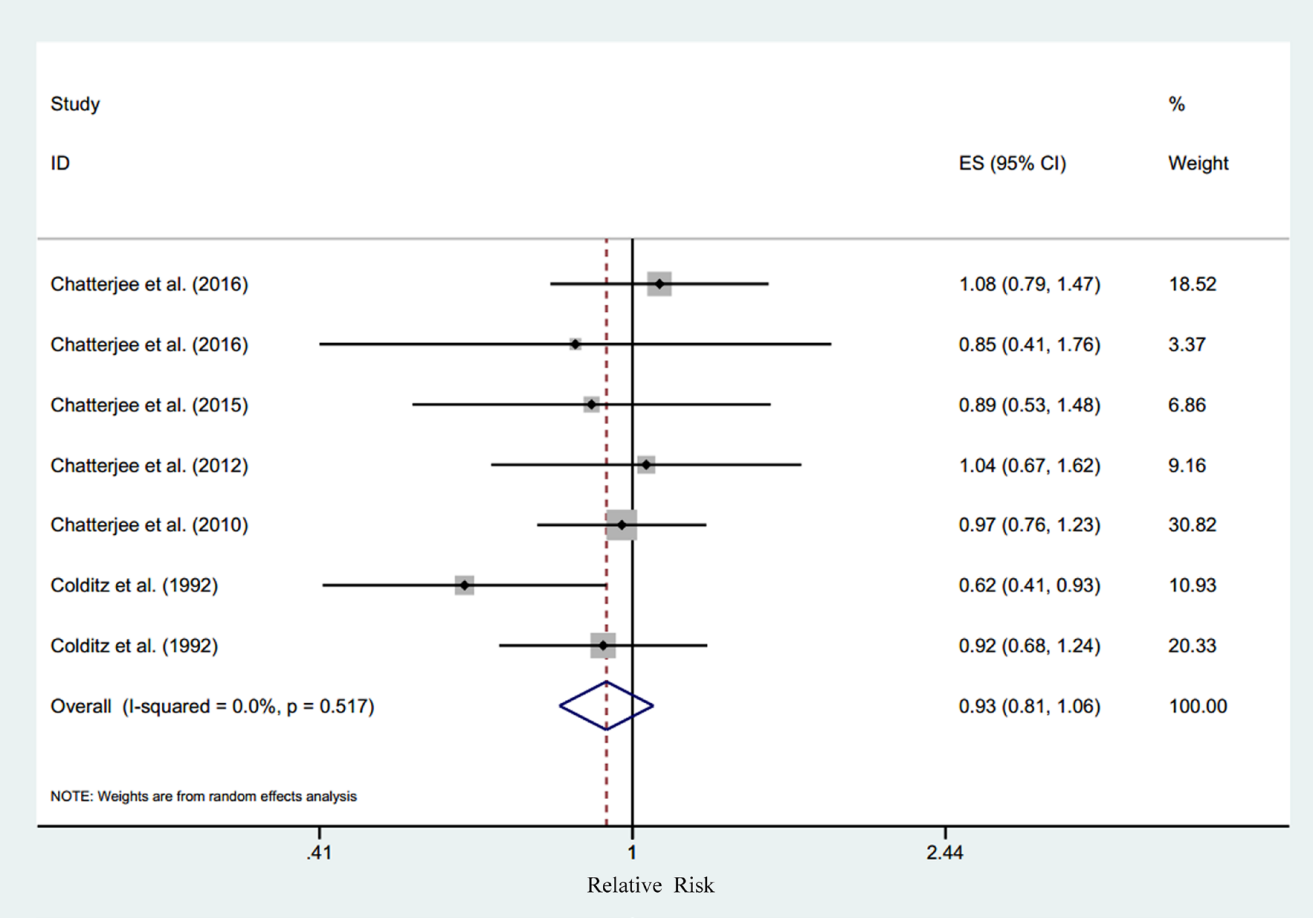
Relative risk of type 2 diabetes according to the highest vs. lowest category of dietary potassium Note: CI = confidence interval, ES = effect size.

**Figure 5:**
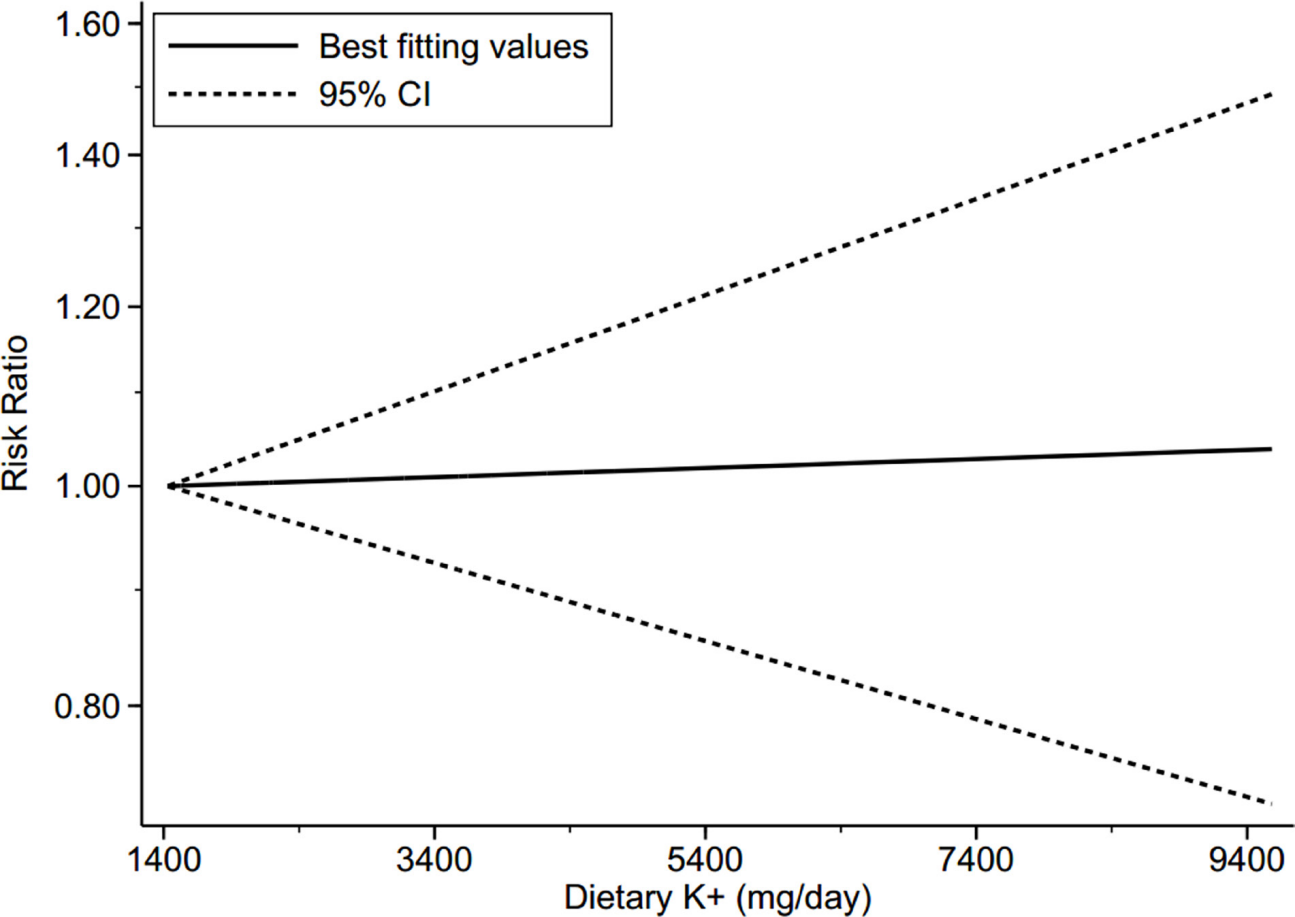
Dose-response relationship between dietary potassium and risk of type 2 diabetes Note: Risk ratio indicates the relative risk of type 2 diabetes. CI = confidence interval, Dietary K^+^ = dietary potassium.

Six studies [9–12, 14, 16] explored the association between dietary potassium and the risk of T2DM, comprising of 112, 125 individuals and 4,573 cases. We failed to find a significant association between dietary potassium and T2DM risk (RR for the highest versus lowest category was 0.93, 95% CI 0.81–1.06, I^2^ = 0.0%, *P*_heterogeneity_ = 0.52, 6 studies) (Figure 4). The sensitivity analysis did not significantly alter the association between dietary potassium and T2DM risk. Meanwhile, there was no significant dose–response relationship between dietary potassium and T2DM risk (RR for every 1000mg increase per day was 1.00, 95% CI 0.96–1.05, goodness-of-fit χ^2^ = 4.53, *P*_goodness-of-fit_ = 0.97, 4 studies). Evidence of a linear association was found (*P*_non-linearity_ = 0.66, 4 studies) (Figure 5).

### Urinary potassium and T2DM

Only three studies [10, 12, 17] were included for urinary potassium and T2DM risk, with 4,376 individuals and 455 cases. On the basis of limited studies, a non-significant pooled risk estimate (RR = 0.83, 95% CI 0.39–1.75, I^2^ = 73.9%, *P*_heterogeneity_ = 0.02, 3 studies) (Supplementary Figure 5) was found for the highest versus lowest category of urinary potassium. The sensitivity analysis did not significantly alter the association between urinary potassium and T2DM risk. There was no statistically significant outcome in random-effects dose-response meta-analysis, with an RR of 1.00 (95% CI 0.95–1.05) per 10 mmol increase in urinary potassium per 24 hours. We found evidence of a linear trend (*P*_non-linearity_ = 0.13, 3 studies) (Supplementary Figure 6).

## DISCUSSION

Our dose-response meta-analysis demonstrated that serum potassium levels were linearly associated with the risk of T2DM, with each 1 mmol/L increase in serum potassium lowering the risk by approximately 17%. Despite the results of the highest versus lowest meta-analysis on serum potassium and the risk of T2DM being statistically non-significant, we observed a trend toward significance. Moreover, a statistically significant inverse association was identified after excluding the two outliers. Heterogeneity appeared to be related to a difference in the average age of subjects. Our study did not detect a significant relationship between dietary or urinary potassium and the risk of T2DM.

Our study is the first meta-analysis to confirm this association quantitatively and is consistent with the results of two previous qualitative reviews [19, 20] which observed lower serum potassium associated with a higher risk of T2DM. Interestingly, both reviews suggested a probable inverse association between dietary potassium and the risk of T2DM, which was inconsistent with the result of our present study.

The effects of thiazide diuretics on the risk of T2DM have been discussed for over half a century. A positive association with increased blood glucose [21, 22] as well as diabetes [23, 24] has been demonstrated. The strong relationship between thiazide-induced hypokalemia and glucose intolerance has been supported by multiple studies [25, 26]. A quantitative analysis [25] of 59 clinical trials suggests the Pearson’s correlation coefficient for the association between glucose and potassium is -0.54 (95% CI = -0.67 to -0.36, *P* < 0.01). These results are supported by another randomized clinical trial of 3790 non-diabetic participants [26], where every 0.5 mmol/L decrease in serum potassium is related to a 45% higher risk of thiazide-induced diabetes.

A large number of experimental studies have been conducted to determine whether experimentally induced hypokalemia is directly associated with glucose metabolism. In a study performed by Rowe et al [27], seven healthy male subjects were subjected to induced potassium deficiency by a low-potassium diet. The glucose-clamp technique test demonstrated that potassium depletion was correlated with the decrease in insulin response (r = 0.78, *p* < 0.05*)*. Further experimental evidence indicates glucose intolerance induced by hypokalemia could be reversed by potassium supply [28–31]. More specifically, potassium supplementation could improve pancreatic β-cell function [32] by increasing insulin levels and sensitivity [33]. Nevertheless, more studies are needed to prove this hypothesis further.

Several animal model studies [34–36] supported the aforementioned conclusions and put forward some enlightening hypotheses about the mechanism of the effects of potassium on glucose tolerance. For example, both the elevation of glucagon and the suppression of insulin were found in response to low potassium [35]. High potassium was found to improve insulin resistance by decreasing plasma renin activity and angiotensin 2 levels [36].

In addition, further studies have concentrated on a molecular mechanism. A meta-analysis of 42, 573 individuals in East Asia [37], showed that single nucleotide polymorphisms (SNPs) of the potassium inwardly-rectifying channel, subfamily J, member 11 (KCNJ11) gene were significantly related to the risk of T2DM. Another study [38] verified that several genetic variations in the KCNJ11 genes, especially E23K polymorphism, were linked to increased T2DM risk. The KCNJ11 encode ATP-dependent potassium channels, important transmembrane proteins expressed in pancreatic β-cells, which are involved with insulin secretion and are also one of the major therapeutic targets of T2DM [39–41]. Potassium levels influence cell depolarization and repolarization with concomitant insulin secretion and release [20]. Taken together, these studies support possible biological or physiological foundations for our results.

The significant linear inverse relationship between serum potassium and T2DM risk may be explainable by the above-mentioned mechanisms. For dietary and urinary potassium, the null associations are probably based on the different measurements of potassium intake. Though food frequency questionnaires (FFQ) and 24h urinary potassium levels are the two most accurate measurements of potassium intake [19], these measurements tend to vary by researcher. Besides, in most included studies, the FFQ was administered only once, and the 24 h urinary potassium levels were calculated from a random urine sample, neither of which are ideal measurements of potassium intake. While serum potassium is regulated by dietary intake and renal excretion, there is no consensus concerning the relationship between dietary potassium and circulating potassium levels. Further studies are needed to explore the precise association between dietary and serum potassium.

Our study has several prominent strengths. Firstly, all included studies are prospective cohorts which result in fewer recall and selection biases. Moreover, we employed the most fully adjusted models to decrease the potential confounding factors. More information could be obtained from the combined use of categorical and dose–response statistical methods.

There are also limitations. First, the number of included studies was limited. Thus, we did not perform subgroup analysis to explore the potential effect modifiers. In addition, several studies have the same first or prominent co-author, the validity of our conclusions may thus be influenced due to the clustering around one author. Second, owing to the nature of observational studies, the residual confounding factors are not fully excluded. Furthermore, dietary potassium was assessed by self-reported FFQs, which might have led to measurement errors. Considering the inconsistent diagnostic criteria of T2DM across studies, both under-reporting and over-reporting are possible. Finally, except for one study performed in Japan [13], other studies are all from North-America and Europe, which limit the generalizability of our results. Future worldwide studies are warranted with use of a consistent definition of T2DM and more reliable measurements of potassium.

In conclusion, our meta-analysis suggests that low serum potassium increases the risk of T2DM in a linear dose-response manner, especially in relatively young individuals. However, neither dietary potassium nor urinary potassium shows any association with the risk of T2DM.

Our results cannot prove causality due to the nature of epidemiologic studies. Future clinical trials are needed to determine if addressing low serum potassium decreases T2DM risk.

## MATERIALS AND METHODS

### Search strategy

Our meta-analysis was conducted following the MOOSE (meta-analysis of observational studies in epidemiology) guidelines [42]. We searched PubMed and EMBASE from inception to January 6, 2017 for relevant studies. Detailed information of search strategy was displayed in the Supplementary Table 2. In addition, a manual search of the references of the included studies and pertinent reviews was conducted. The language was restricted to English and Chinese. We contacted the original authors for extra information through e-mails when it was necessary.

### Study selection

Citations were independently reviewed by two investigators (Y.P. and A.W.). We included prospective cohort studies on the association between potassium measurements (serum, dietary or urinary potassium) and the risk of T2DM in the general population that reported the adjusted risk estimates (risk ratios, hazard ratios or odds ratios) and corresponding 95% confidence intervals (CIs). The most recent article was considered for inclusion when multiple articles derived from the same cohort. We excluded the study of gestational diabetes, new-onset diabetes after transplantation and type 1 diabetes. A consensus was reached by discussion in case of any discrepancy.

### Data extraction and quality assessment

A standard extraction form was used in the process of data extraction by two independent investigators (K.L. and Q. M.). Individual study was extracted with following information: first author, publication year, study location, age range, mean age, gender, sample size, incident T2DM cases, follow-up duration, categories of potassium, diagnostic criteria of T2DM, adjusted risk estimates and corresponding 95% CIs and adjustment variables.

We adopted the Newcastle–Ottawa Scale, which is frequently selected for observational studies in meta-analyses, to evaluate the methodological quality of the selected studies. The total score ranges from 0 to 9 based on selection of participants and exposure, comparability on confounders, evaluation of outcomes, and sufficient follow-up. The total score exceeding six is regarded to be high-quality.

### Statistical method

We calculated summary risk estimates from each individual study using a random-effects model. Hazard ratio, odds ratio and risk ratio were directly regarded as equivalents. For studies [12, 14] with mg kcal^−1^or mg kJ^−1^ day^−1^as the unit of dietary potassium, we unified the unit to mg day^−1^ by multiplying average amount of daily energy intake provided in the study. The total 24h urinary potassium was approximately estimated by multiplying the spot urinary potassium (mmol/L) with the mean urinary volume per day if it was not available in the study [10]. For several studies [9, 11–14, 17] whose reference category was not the lowest category, the risk estimates were converted using the method proposed by Hamling et al [43]. Both the Cochran’s Q statistic [44] (P < 0.10 suggesting statistically significant) and the I^2^ statistic (I^2^ > 75.0%, 50.0–75.0% and < 50% indicating substantial, moderate and low heterogeneity, respectively) were calculated for evaluating heterogeneity across studies.

A random-effects dose-response meta-regression model described by Orsini et al [45] was carried out on the basis of specific potassium level, distribution of cases and person-years, and adjusted risk estimates and 95% CIs. On the condition that potassium category was provided as range, the midpoint of each range was designated as the assigned dose. When the highest range was open-ended, the width of the adjacent interval was added to the highest limit specified to obtain the assigned dose. When the lowest range was open-ended, we estimated the assigned level by subtracting half of the width of the adjacent interval from the lowest limit specified [46]. If the number of person-years by potassium level was not directly presented in original articles [9–12], these required data were approximately calculated by multiplying the number of participants in each level with the mean follow-up duration. We used restricted cubic spline function [18, 45] with 3 knots at the 10th, 50th and 90th percentiles to explore the potential non-linear dose–response association. A *P*_non-linearity_ was identified by testing the null hypothesis that the estimated value of the second spline was equal to zero. Two studies [14, 16] were included in the highest versus the lowest meta-analysis to examine the robustness of the results further. Due to the Nurses’ Health Study (NHS) [16] was stratified by body mass index (BMI), both the two subgroups (BMI > 29 kg/m^2^ and BMI < 29 kg/m^2^) were used for comparing the highest versus lowest category of dietary potassium.

To ensure the stability of the results and to explore the possible sources of heterogeneity, sensitivity analyses were performed by omitting one study in turn. Due to the limited studies, subgroup analysis was not conducted. Begg’s and Egger’s tests were adopted to assess publication bias. Funnel plots were also mapped [47]. We carried out all data analyses using STATA software (version12.0, StataCorp LP, College Station, Texas, USA). Statistical significance level was assigned at *P* < 0.05 under a two-sided test.

## SUPPLEMENTARY MATERIALS FIGURES AND TABLES



## Abbreviations

BMI: Body Mass Index
CIs: confidence intervals
FFQ: food frequency questionnaires
KCNJ11: potassium inwardly-rectifying channel, subfamily J, member 11
RR: relative risk
T2DM: type 2 diabetes mellitus
