# Supplementary material for: Potassium measurements and risk of type 2 diabetes: a dose-response meta-analysis of prospective cohort studies
Source: Oncotarget. 2017 Oct 11;8(59):100603–13. doi: 10.18632/oncotarget.21823 (PMC5725047; doi:10.18632/oncotarget.21823)
Supplement: Supplementary file 1 [file oncotarget-08-100603-s001.pdf]

Potassium measurements and risk of type 2 diabetes: a dose-response meta-analysis of prospective cohort studies

SUPPLEMENTARY MATERIALS

Supplementary Table 1: The results of quality assessment for cohort studies

Study	Selection				Comparability	Outcome			Total Score
	Representativeness of exposed cohort ☆	Selection of non-exposed cohort ☆	Exposure ascertainment ☆	No DM at enrollment ☆	Comparable on confounders ☆☆	Outcome Assessment ☆	Adequate follow-up (≥ 10y) ☆	Loss to follow-up rate (≤ 20%) ☆	
Chatterjee et al. [9] 2016	☆	☆	☆	☆	☆ ☆			☆	7
Chatterjee et al. [10] 2016	☆	☆	☆	☆	☆ ☆	☆			7
Chatterjee et al. [11] 2015	☆	☆	☆	☆	☆ ☆	☆	☆	☆	9
Chatterjee et al. [12] 2012	☆	☆	☆	☆	☆ ☆	☆	☆	☆	9
Heianza et al. [13] 2011		☆	☆	☆	☆ ☆				5
Chatterjee et al. [14] 2010	☆	☆	☆	☆	☆ ☆			☆	7
Hu et al. [17] 2005	☆	☆	☆	☆	☆ ☆	☆	☆	☆	9
Colditz et al. [16] 1992		☆	☆	☆	☆ ☆				5

Supplementary Table 2: Search Strategy for PubMed and EMBASE databases

Search strategy for PubMed database (from its inception to January 6, 2017)

No.	Search strategy	Items found
#1	(potassium, dietary [MeSH Terms]) OR potassium [MeSH Terms] OR potassium [Title/Abstract]	180315
#2	(diabetes mellitus [MeSH Terms]) OR diabetes [Title/Abstract]) OR diabetic [Title/Abstract]) OR DM [Title/Abstract] OR “glucose intolerance” [Title/Abstract]) OR T2DM [Title/Abstract] OR T2D [Title/Abstract]	576858
#3	English [Language] OR Chinese [Language]	22536747
#4	#1 AND #2 AND #3	4131
#5	Review [Publication Type] OR Letter [Publication Type] OR Editorial [Publication Type] OR Published erratum [Publication Type]	3628202
#6	#4 NOT #5	3421

Search strategy for EMBASE database (from its inception to January 6, 2017)

No.	Search strategy	Items found
#1	‘potassium intake’/exp OR ‘potassium’/exp OR potassium:ab,ti	214, 569
#2	‘diabetes mellitus’/exp OR diabetes:ab, ti OR diabetic:ab, ti OR DM:ab,ti OR ‘glucose intolerance’:ab, ti OR T2DM:ab, ti OR T2D:ab,ti	934, 265
#3	English:la OR Chinese:la	26, 674, 779
#4	#1 AND #2 AND #3	7, 802
#5	review:it OR letter:it OR editorial:it OR erratum:it	3, 804, 014
#6	#4 NOT #5	6, 553

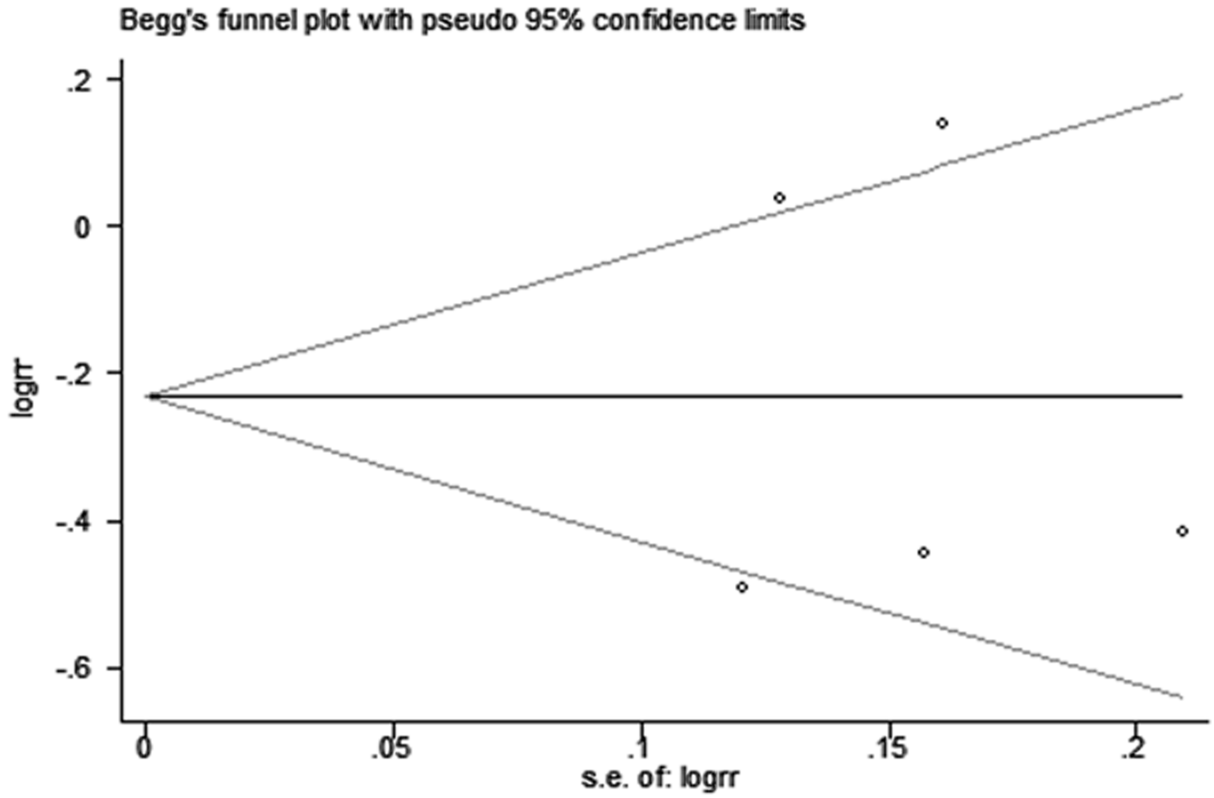

Supplementary Figure 1: Funnel plot to explore publication bias on serum potassium and risk of type 2 diabetes.

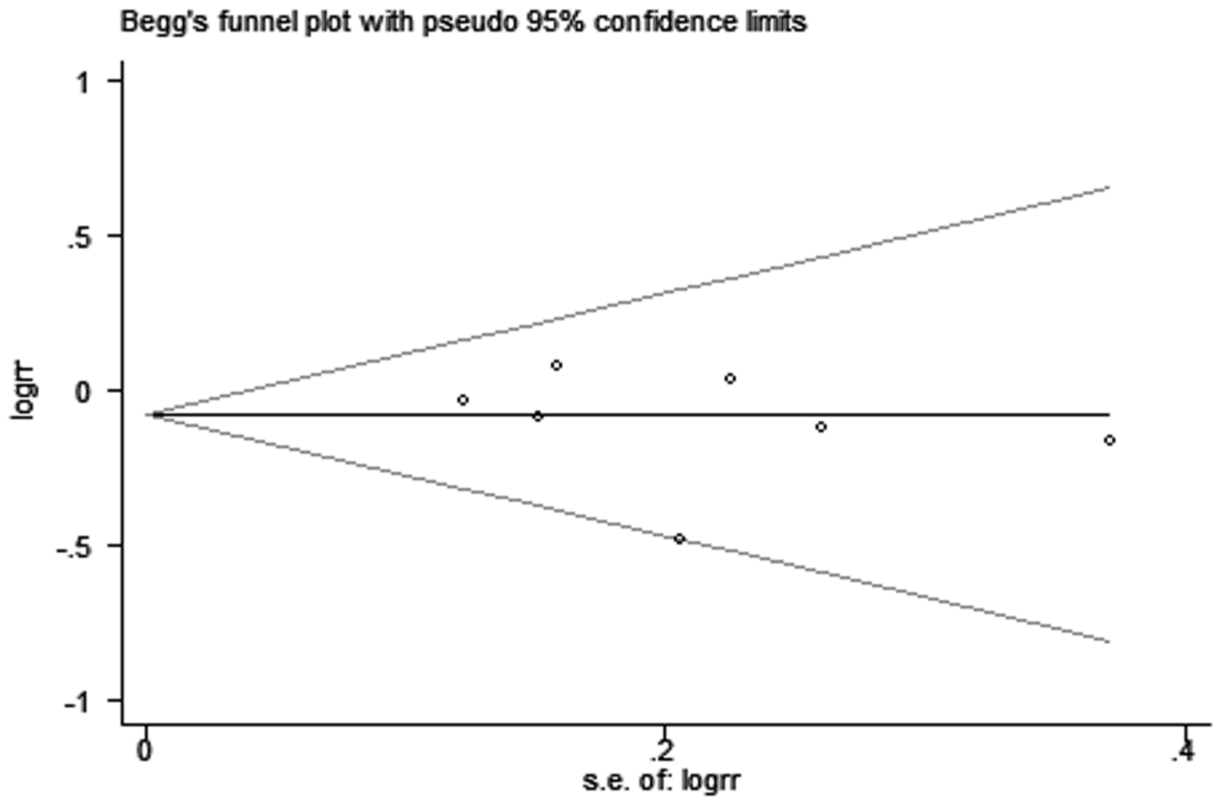

Supplementary Figure 2: Funnel plot to explore publication bias on dietary potassium and risk of type 2 diabetes.

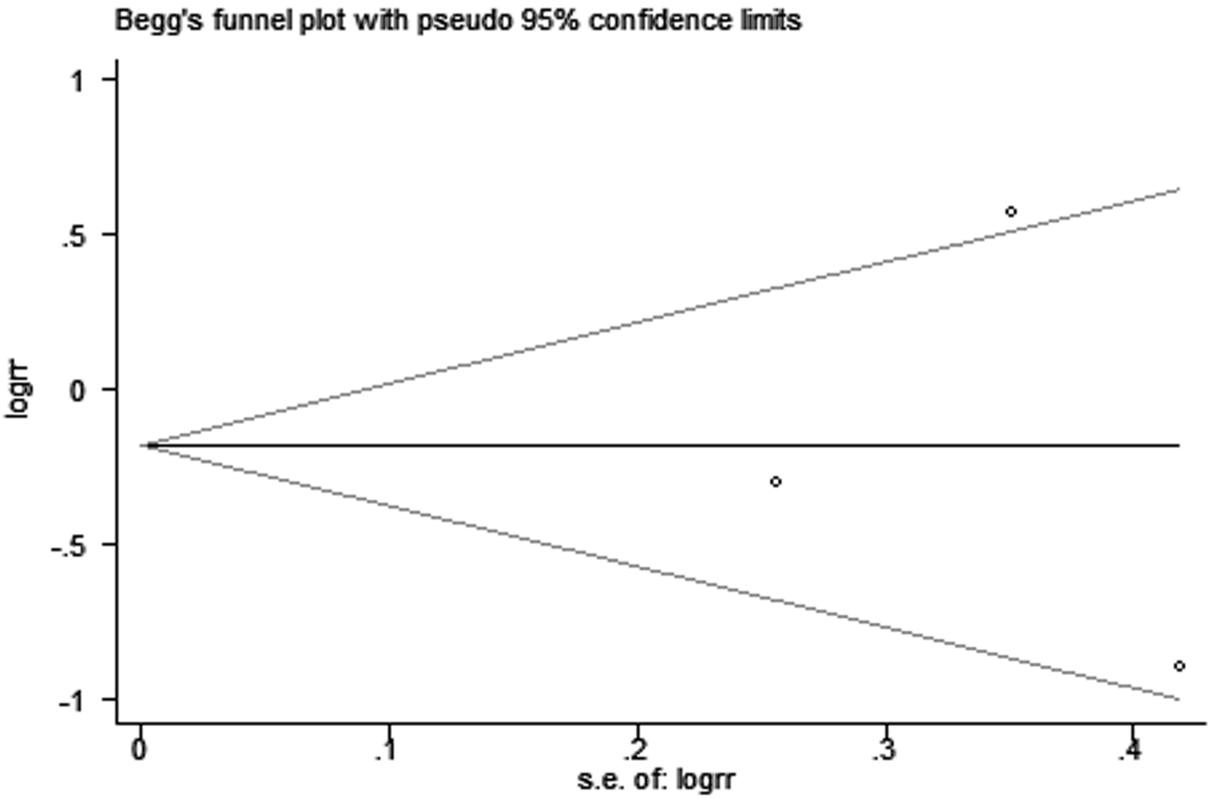

Supplementary Figure 3: Funnel plot to explore publication bias on urinary potassium and risk of type 2 diabetes.

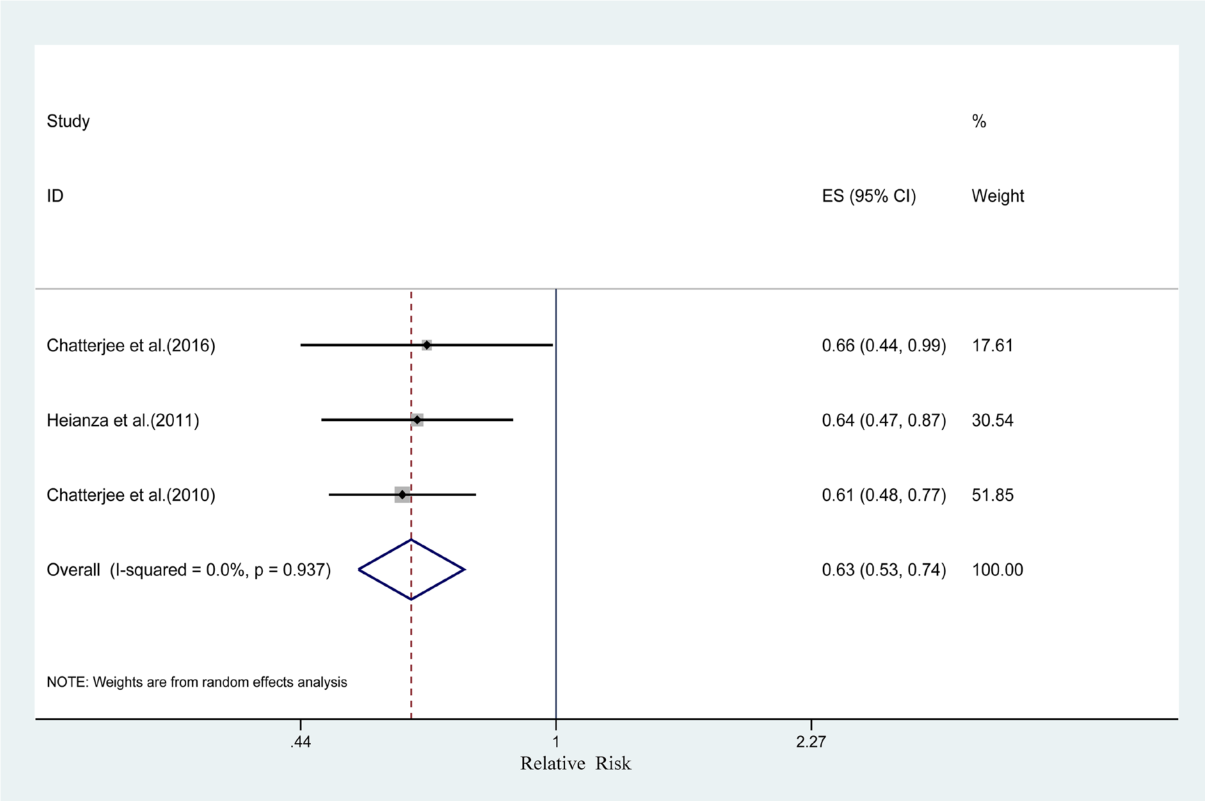

Supplementary Figure 4: Relative risk of type 2 diabetes according to the highest vs. lowest category of serum potassium. Note: The relative risk was pooled by using a random-effects meta-analysis by ignoring two studies with much older mean age. CI = confidence interval, ES = effect size.

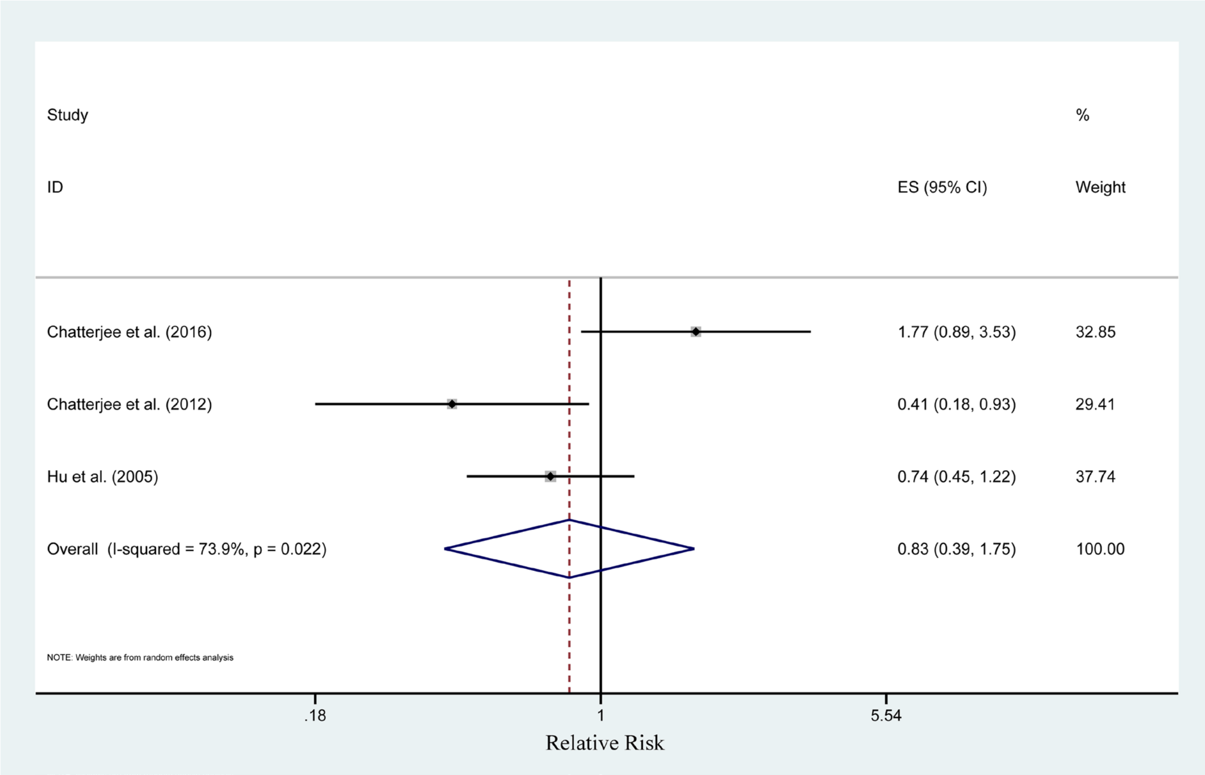

Supplementary Figure 5: Relative risk of type 2 diabetes according to the highest vs. lowest category of urinary potassium. Note: CI = confidence interval, ES = effect size.

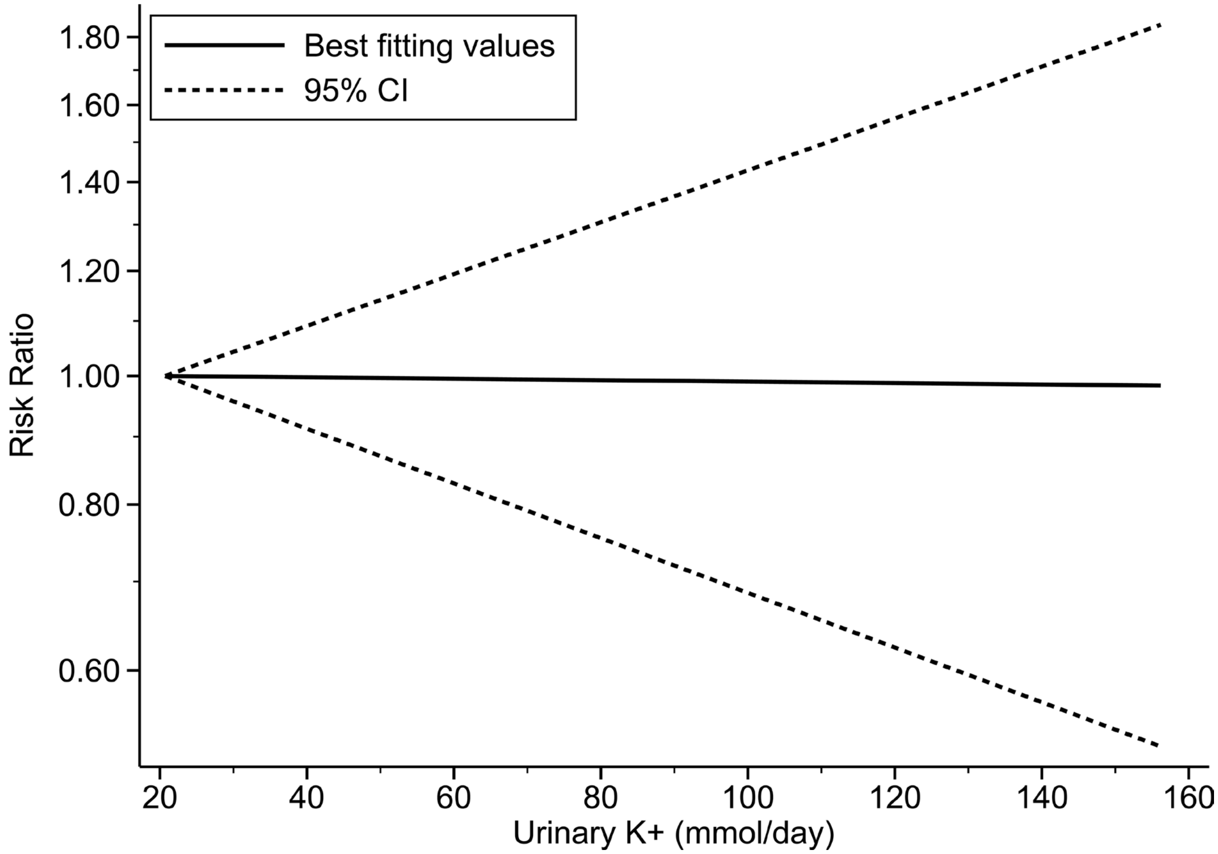

Supplementary Figure 6: Dose-response relationship between urinary potassium and risk of type 2 diabetes. Note: Risk ratio indicates the relative risk of type 2 diabetes. CI = confidence interval, Urinary K^+ = urinary potassium.
